# Effects of remote group-based exercise on physical activity and well-being in postpartum women: a randomized controlled trial

**DOI:** 10.1186/s12966-026-01897-x

**Published:** 2026-02-25

**Authors:** Yumi Nomura, Mako Fukano, Kosuke Kashiwabara, Megumi Haruna

**Affiliations:** 1 Faculty of Creative Engineering, Chiba Institute of Technology, 2-1-1, Shibazono, Narashino-Shi, Chiba 275-0023 Japan; 2 College of Engineering, Shibaura Institute of Technology, 307 Fukasaku, Minuma-Ku, Saitama-Shi, Saitama, 337-8570 Japan; 3 Data Science Office, Clinical Research Promotion Center, The University of Tokyo Hospital, 7-3-1 Hongo, Bunkyo-Ku, Tokyo 113-8655 Japan; 4 Department of Midwifery and Women’s Health, Division of Health Sciences and Nursing, Graduate School of Medicine, The University of Tokyo, 7-3-1 Hongo, Bunkyo-Ku, Tokyo, 113-0033 Japan

**Keywords:** Physical activity, Exercise, Mother, Postpartum, Quality of life, Randomized controlled trial, Web-based, Online

## Abstract

**Background:**

Postpartum women frequently experience declines in physical activity (PA) resulting from lifestyle changes, caregiving demands, and physical recovery, which increases the risk of long-term physical and mental health issues. While behavioral strategies can promote PA, few interventions address the lifestyle-related barriers specific to postpartum women using objective PA measures, and remote, group-based approaches remain underexplored. This study evaluated the efficacy of an 8-week remotely delivered, group-based PA intervention in increasing objectively measured PA, exercise-related self-efficacy, and psychosocial well-being among postpartum women.

**Methods:**

In this web-based, two-arm randomized controlled trial, 175 postpartum women (2–6 months postpartum) in Japan were allocated to either an intervention (*n* = 89) or waitlist control group (*n* = 86). The intervention combined weekly instructor-led online group sessions with a structured home-based exercise program, incorporating behavioral strategies grounded in self-determination and social cognitive theories. The primary outcome was daily moderate-to-vigorous PA (MVPA) measured via triaxial accelerometers. The secondary outcomes included daily step counts, health-related quality of life (HRQoL; Short Form-12 Health Survey version 2), sense of coherence (SOC; Sense of Coherence Scale), and exercise self-efficacy (decisional balance for exercise). Analyses used generalized estimating equations adjusting for baseline values and age.

**Results:**

Retention was 98%–99%, with 94% attending at least four of six classes. Compared with controls, the intervention significantly increased MVPA by 5.97 min/day (95% confidence interval [CI]: 1.34, 10.60; *p* = 0.012) and daily steps by 576 (95% CI: 73, 1079; *p* = 0.025). SOC increased by 4.14 points (95% CI: 1.70, 6.58; *p* < 0.001) and exercise self-efficacy increased (balance score difference: 2.74; 95% CI: 0.71, 4.78; *p* = 0.008), mainly because of reduced perceived barriers. No significant changes in HRQoL were observed.

**Conclusions:**

This remote, group-based PA intervention, designed to accommodate the lifestyle demands of the postpartum period, effectively increased PA and enhanced psychosocial resources in postpartum women. By fostering self-efficacy, peer support, and accessible home-based participation, this program may support both short- and long-term physical and mental health. These findings highlight the potential of scalable online PA programs to overcome common postpartum barriers.

**Trial registration:**

University Hospital Medical Information Network Clinical Trials Registry (UMIN-CTR): UMIN000053478, registered 31 January 2024.

**Supplementary Information:**

The online version contains supplementary material available at 10.1186/s12966-026-01897-x.

## Background

Postpartum women often face considerable challenges in maintaining adequate levels of physical activity (PA), despite its well-established physical and psychological health benefits [1–3]. The early postpartum period is characterized by significant lifestyle changes, such as sleep disruption, caregiving responsibilities, and physical recovery from childbirth. These changes frequently act as barriers to regular participation in PA [4]. Consequently, many postpartum women experience a marked decline in moderate-to-vigorous physical activity (MVPA) [5, 6], with the majority failing to meet national or international PA recommendations during this period [7, 8]. Current guidelines, including those from the World Health Organization, recommend that pregnant and postpartum women engage in at least 150 min of MVPA per week [9–11], underscoring the public health relevance of this decline. Insufficient PA after childbirth is associated with increased risks of long-term health issues, including obesity [12], cardiovascular disease [13], and poor mental health outcomes such as postpartum depression [14]. Therefore, promoting PA among postpartum women is a critical public health priority.

Numerous interventions have been developed to promote PA during the postpartum period; however, evidence regarding their effectiveness remains inconclusive. A systematic review and meta-analysis by Schulz et al. [15] evaluated the effects of exercise interventions on postpartum PA and cardiorespiratory fitness. Although some improvements were observed in fitness outcomes, they concluded that most interventions had limited effects on objectively measured PA. The authors also identified a lack of behavioral strategies and insufficient intervention intensity as potential explanations for these modest outcomes. By contrast, Lim et al. [16] emphasized the effectiveness of behavioral strategies in changing PA and eating behaviors among postpartum women. Their meta-analysis found that interventions incorporating techniques such as self-monitoring, goal setting, feedback, and credible sources led to significant improvements in PA, although none of these behavioral strategies explained the heterogeneity in PA outcomes. These findings suggest that while behavioral strategies are useful in promoting PA, it is insufficient simply to adopt strategies designed for the general population. Rather, it is essential to consider the unique barriers and facilitators specific to postpartum women when designing interventions.

Previous studies have identified a wide range of individual, interpersonal, and environmental factors that influence PA among postpartum women. For instance, lack of time, fatigue, low motivation, limited access to childcare, and inadequate social support have been consistently reported as key barriers [17–19]. Conversely, social support, particularly from partners and peers, as well as a sense of competence and autonomy, have been shown to facilitate PA engagement [20–22]. These findings underscore the importance of incorporating both intrapersonal and interpersonal components into intervention designs.

Group-based interventions are widely used to promote health and support behavior change, including participation in PA. As postpartum women are less likely to engage in PA because of competing demands (e.g., caregiving duties, domestic responsibilities) and a variety of individual and environmental barriers, social support mechanisms such as interaction and communication with peers in the same life stage and personalized, progressive activities offered in group-based interventions may help women overcome these challenges [4, 23, 24]. Additionally, because social support was emphasized in the intervention and has been shown to be important for preventing depression in the late pregnancy and early postpartum periods [25], the effect of the intervention on social support was also explored. Peralta et al. [26] conducted a systematic review of group-based PA interventions targeting postpartum women with children aged 0–5 years. Analyzing six randomized controlled trials (RCTs), they found that group-based programs were moderately successful in increasing *self-reported* PA levels. However, few studies included objective assessments of PA; only one trial used objective measures, and overall, group-based interventions were not successful in increasing *objectively measured* PA levels [27]. Additionally, most interventions were delivered face-to-face, which may limit accessibility for postpartum women who are balancing childcare and household responsibilities.

Despite their potential, few high-quality RCTs have evaluated the effectiveness of remote, group-based PA interventions for postpartum women using objective outcome measures such as accelerometry. Moreover, existing interventions rarely address both physical and psychosocial outcomes—such as health-related quality of life (HRQoL), sense of coherence (SOC), and self-efficacy for exercise—which are critical for sustaining behavioral change during the postpartum period. SOC has been reported to be a protective factor against stress and depressive symptoms and is considered a salutogenic resource that enhances coping capacity and promotes positive health behaviors [28, 29]. Likewise, self-efficacy is a key determinant of PA engagement [30], especially during pregnancy and the postpartum period [31, 32].

To address these gaps, the present study aimed to evaluate the efficacy of an 8-week remotely delivered, group-based PA intervention designed for postpartum women as a strategy to enhance self-efficacy for PA and promote sustained engagement in PA behaviors. The intervention incorporated supervised online group sessions and a structured home-based exercise phase grounded in self-determination theory [33] and social cognitive theory [34], with the goal of improving both objectively measured PA levels and psychological well-being. We hypothesized that participants randomized to the PA intervention would report greater increases in MVPA, HRQoL, SOC, and self-efficacy for exercise compared with participants randomized to the usual care condition.

## Methods

### Study design

**Fig. 1:**
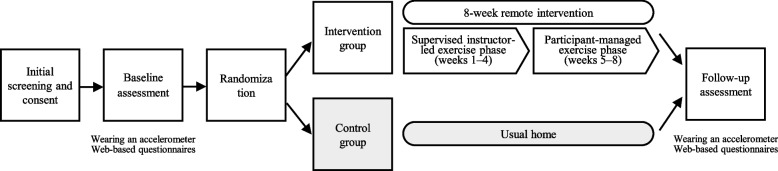
Study design

The study design is presented in Fig. 1. The present study was an 8-week, two-arm, parallel-group, web-based RCT comparing the efficacy of an intervention arm consisting of exercise groups that used videoconferencing and a waitlist control. The study design, conduct, and reporting were performed in accordance with the Consolidated Standards of Reporting Trials (CONSORT) guidelines [35] (CONSORT checklist Additional file 1, TIDieR checklist Additional file 2). Prior to study enrollment, all participants provided electronic informed consent for participation. The goal of the PA intervention was to increase MVPA to 23 metabolic equivalent (MET) hours per week, which is consistent with the standards of the Japanese Ministry of Health, Labour and Welfare (Review Committee on the Revision of Physical Activity Reference for Health Promotion 2013) [36]. The 8-week intervention duration was determined primarily based on evidence from group-based PA interventions for postpartum women. A systematic review reported intervention durations ranging from 1 to 9 months, with mixed effects on PA outcomes [26]. Notably, an 8-week randomized controlled trial demonstrated significant improvements in PA using a two-phase design consisting of an initial 4-week intensive group-based intervention followed by a 4-week home-based self-structured exercise phase [37]. This evidence informed the design and duration of the present intervention, with additional consideration given to feasibility and participant burden during the postpartum period.

### Participants

The inclusion criteria for the present study were: (a) living in Japan; (b) > 20 years of age; (c) ability to speak and read Japanese; (d) between 2 and 6 months postpartum; and (e) had completed a 1-month postpartum checkup with a healthcare provider. Women with restricted PA by a physician or other healthcare provider or those experiencing psychiatric disorders were excluded. G*Power (version 3.1.9.7; Heinrich Heine University Düsseldorf, Düsseldorf, Germany) was used to calculate the sample size needed to identify differences in primary outcomes between the two groups. The sample size was 140 participants with an alpha error of 0.05 and a power of 0.8, referring to an effect size of 0.48 from a previous study [37]. Assuming a dropout rate of 15%, we aimed to recruit at least 166 participants (83 in each group).

### Recruitment, assignment, randomization, and blinding

Participants were recruited between January 2024 and January 2025. To support information dissemination, a dedicated website and Instagram account outlining the study details were created. Participants were recruited using: 1) social media advertisements (Instagram and Facebook) that included links to the study website and Instagram account; and 2) fliers distributed at eight obstetric-related medical facilities in urban areas of Japan (Tokyo, Kanagawa, Chiba, and Osaka). Additionally, all enrolled participants were encouraged to share the study website or Instagram account with individuals they thought might be interested. Participants who were interested in the study were instructed to respond via the online recruitment form. Once written informed consent was obtained and eligibility confirmed by the researchers, the baseline questionnaires were sent via email, and a triaxial accelerometer was mailed to each participant. Participants also received an activity log and a self-addressed, stamped envelope to return the accelerometer and log after the 7-day wear period.

Group assignment was performed using block randomization (block size 4, 1:1 ratio) stratified by parity (primiparous or multiparous), with a computerized random number table. A research assistant who was not involved in follow-up assessments conducted the randomization. Participants in both groups took part in the same PA program at different times; however, the specific differences between the groups were not disclosed. Although participants were aware that the study focused on PA, they were not informed of their group allocation, and the coresearchers were careful not to disclose this information—thus maintaining blinding of the participants to some extent. However, complete blinding of the participants and instructors was not possible owing to the nature of the intervention. Data entry personnel were blinded to group allocation, and the data analysis was conducted by a non-blinded assessor.

### Interventions

The postpartum PA program was developed by exercise specialists and a supervising midwife, and was explicitly informed by self-determination theory [33] and social cognitive theory [34]. In accordance with self-determination theory, the intervention was designed to support autonomy, competence, and relatedness. Autonomy was promoted by encouraging participants to select preferred activities and set individualized goals, particularly during the later, participant-managed portion of the program. Competence was supported through progressive exercise instruction, individualized feedback on PA levels, and goal review. Relatedness was fostered through regular group-based online sessions that emphasized peer interaction and mutual support. Consistent with social cognitive theory, the intervention incorporated strategies to enhance self-efficacy and behavioral regulation, including goal setting, self-monitoring, behavioral feedback, problem-solving, and exposure to credible sources (i.e., certified instructors). These strategies have been associated with significant improvements in the health behaviors of postpartum women [16]. The intervention consisted of two sequential phases (Fig. 1, Additional file 3):

1. A supervised, instructor-led exercise phase with weekly remote group sessions (weeks 1–4), and
2. A participant-managed exercise phase with biweekly remote group sessions (weeks 5–8).

Participants in the control group were offered the same program after outcome measurements were completed.

The periodized training plan included a variety of exercises aimed at improving posture, strength, flexibility, balance, abdominal recovery, cardiorespiratory fitness, and pelvic floor muscle function. The content was informed by PA guidelines for the pregnancy and postpartum periods from the American College of Obstetricians and Gynecologists [10], Sports Medicine Australia [11], and the International Olympic Committee [38].

#### Supervised instructor-led exercise phase (Phase 1)

The supervised instructor-led exercise phase included weekly remote group exercise sessions conducted by three certified fitness instructors with expertise in pregnancy and postpartum fitness. All instructors completed a 6-h training session specific to this intervention, engaged in independent review of the program content, and underwent practical skill checks conducted by the principal investigator prior to delivering the sessions. Each session was facilitated by an instructor and supported by a research assistant. The research assistant confirmed participant attendance, managed the videoconferencing platform operations, and, when participants were unable to attend their regular session, proposed attendance at an alternative session offered on a different weekday. After randomization, participants selected a weekday morning session that suited their availability. Each group consisted of six to 10 participants. Participants and instructors joined the sessions via a secure videoconferencing URL on Zoom (version 6.5.9; Zoom Video Communications, Inc., San Jose, CA, USA), shared only with involved parties. Each 80-min session followed this structure:

1. Greeting and warm-up (10 min)
2. Brief lecture on postpartum physical changes and the importance of PA (5 min)
3. Muscular stretching and strengthening (40 min)
4. Rhythmic dancing (5–10 min)
5. Cooldown and small group communication (15 min)

Communication among participants was emphasized as it contributes positively to the continuity of PA and maternal mental health. To enhance self-awareness, participants received individualized feedback, including their baseline step counts and activity levels, via email before the fourth session. Participation with or without an infant was allowed, and mothers were encouraged to feed or attend to their infants freely during the class. To ensure intervention fidelity, the principal investigator periodically reviewed session recordings to verify adherence to the intervention protocol and provide feedback to instructors as needed. Participant safety was monitored throughout the intervention. At each group exercise session, the instructor verbally asked participants about their physical condition (e.g., “Are you feeling well?”) and confirmed whether any discomfort, pain, or other health concerns occurred during or after the session. In addition, participants were encouraged to report any adverse events voluntarily via email throughout the study period. No additional systematic surveillance procedures were implemented.

#### Participant-managed exercise phase (Phase 2)

The participant-managed exercise phase began immediately after the supervised phase and continued for 4 weeks. In contrast to Phase 1, this phase emphasized participant autonomy, with women independently planning and performing exercise activities, incorporating exercises learned during the supervised instructor-led exercise phase or engaging in other preferred activities.

Participants were:

1. Supported in setting individualized goals and planning home-based activities,
2. Recommended to use freely available exercise videos on YouTube (Additional file 4), which were regularly updated and shared via a smartphone communication app (LINE; LINE Corporation, Tokyo, Japan),
3. Asked to record the type, duration, and intensity of their PA in a log sheet, and
4. Encouraged to send regular motivational and reminder messages via the communication app to sustain engagement and adherence.

Additionally, to maintain engagement, 80-min remote group sessions were held biweekly. These sessions provided opportunities to:

- Review progress, feedback, and praise,
- Share experiences with instructors and peers,
- Revisit and reinforce previously introduced exercises,
- Promote mutual support and collaborative problem-solving, and
- Receive guidance in regard to modifying their exercise plans as needed.

#### Primary outcome

Outcome measures were assessed at two time points: baseline (between 2 and 6 months postpartum) and follow-up (8 weeks after the program; between 4 and 8 months postpartum). The questionnaire included items on demographic characteristics, HRQoL, SOC, and self-efficacy for exercise. Follow-up data collection ended in April 2025. The following three scales were evaluated.

### Physical activity

The primary outcome, average MVPA per day, was assessed using a triaxial accelerometer (Active Style Pro HJA-750C; Omron Healthcare, Kyoto, Japan), which has been shown to have acceptable validity and reliability for assessing PA and sedentary time in adult populations [39, 40]. Participants wore the accelerometer for 7 days on the waist with a clip during waking hours, except when engaging in water-based activities (e.g., bathing, swimming). The normal frequency filter was selected and the epoch length was 60 s. The accelerometer data were processed using a macro program (ver. 1.0) developed and distributed by the Japan Physical Activity Research Platform [41]. Non-wear time was defined as ≥ 60 consecutive minutes of zero counts, which correspond to an estimated metabolic equivalent (MET) value < 1 on the accelerometer. To avoid the inclusion of non-wear periods such as potential motion during mailing, participants were instructed to record the exact times and dates of accelerometer wear in an activity log. Only data recorded during the logged wear period were included in the analysis. A valid day was defined as at least 10 h of accelerometer wear, and participants were required to have at least 4 valid days [42]. As many participants were on parental leave, data from all selected days were analyzed regardless of whether they were weekdays or weekends. Prior to analysis, distributions of accelerometer-measured PA outcomes were inspected using histograms and boxplots for baseline, follow-up, and change scores. No extreme outliers were identified in the change scores, and therefore no accelerometer-derived PA data were excluded as outliers prior to analysis. Physical activity intensity was classified using MET-based cut points derived from the proprietary algorithm of the Active Style Pro. The device estimates activity intensity in METs using a validated triaxial accelerometer algorithm with a gravity-removal classification method developed for adult populations [39, 40]. Based on this algorithm, sedentary behavior was defined as ≤ 1.5 METs, light physical activity as > 1.5 to < 3.0 METs, moderate physical activity as ≥ 3.0 to < 6.0 METs, and vigorous physical activity as ≥ 6.0 METs, consistent with previous studies using the Active Style Pro [43]. Moderate and vigorous activity were combined to define MVPA (≥ 3.0 METs).

#### Secondary outcomes

The following three scales were assessed using web-based questionnaires.

#### HRQoL

The Short Form-12 Health Survey version 2 (SF-12v2) is a widely used standard measurement tool for HRQoL that utilizes a self-administered questionnaire [44]. The SF-12v2 is a shortened version of the Short Form-36 Health Survey (SF-36). The SF-36 and SF-12v2 are the two most frequently used measures of HRQoL for pregnant and postpartum conditions [45]. The SF-36 and SF-12v2 have both been translated into Japanese and offer confirmed validity and reliability in Japanese populations [46]. Items on the SF-12v2 are summarized into two weighted scales—the physical component summary (PCS) and the mental component summary (MCS)—designed to assess physical and mental well-being, respectively. Each is scored to have a mean of 50 and a standard deviation (SD) of 10 in the Japanese population, with lower scores indicating higher levels of impairment.

#### Sense of coherence

SOC was measured using the 13-item Sense of Coherence Scale (SOC-13), which reflects the ability to cope with stress and is central to the theory of salutogenesis. The SOC-13 was treated as a unidimensional model based on Antonovsky [47] and Frenz et al. [48]. A Japanese version of the SOC-13 that consists of 13 items, each scored from 1 to 7, with total scores ranging from 13 to 91, has also been developed [49, 50].

#### Self-efficacy for exercise

The perceived positive (pros) and negative aspects (cons) of exercise were assessed based on decisional balance for exercise, including a 10-item pros scale and a 10-item cons scale [51]. Examples of items in the pros scale were “Regular exercise would help me relieve tension” and “It would be easier for me to perform routine physical tasks if I exercised regularly”. Examples of items in the cons scale were “Regular exercise would take too much of my time” and “I would have less time for my family and friends if I exercised regularly”. The participants rated the extent to which they agreed or disagreed with each item on a five-point Likert-type scale ranging from 1 (strongly disagree) to 5 (strongly agree); good 2-week test–retest reliability (pros: r = 0.80; cons: r = 0.77) and internal consistency (pros and cons scales: α = 0.84) have been reported [52]. All items were subsequently summed to form a single item for pros and cons and dichotomized into high and low categories using a median split.

### Statistical analysis

All statistical analyses were conducted using SPSS (version 28.0; IBM SPSS Japan, Inc., Tokyo, Japan). Demographic and baseline characteristics were reported for participants across the two groups as means (SDs) for continuous variables and percentages (counts) for categorical variables. The equivalence of the control and intervention groups was tested at baseline using an unpaired *t*-test and the chi-square test.

Generalized estimating equation (GEE) analyses [53] were used to compare the groups with outcome data at 8 weeks postintervention in MVPA, number of steps, and psychometric scales (SF-12v2, SOC-13, decisional balance for exercise) scores adjusting for baseline values and age. In each model, the postintervention value was used as the dependent variable, group (intervention vs. control) as independent variables, and age and baseline values as covariates. The analyses were conducted on an intention-to-treat basis using data from all participants who completed the baseline assessment (*n* = 175). However, participants with missing follow-up data (*n* = 2, 1%) were excluded, and a complete-case analysis was subsequently performed on the remaining 173 participants with both baseline and follow-up data. All *p*-values were two-sided, with *p*-values < 0.05 considered to indicate statistical significance.

## Results

### Recruitment and retention

**Fig. 2:**
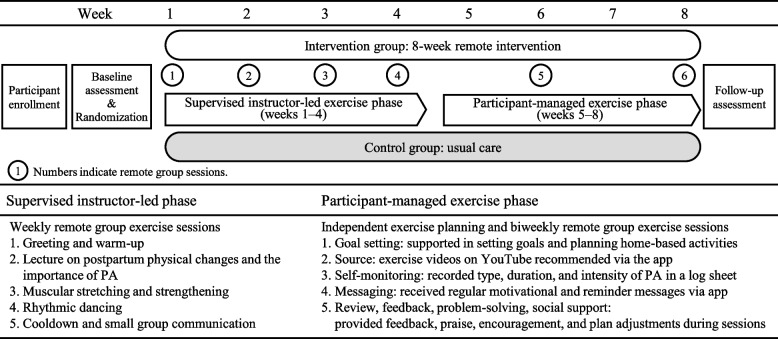
Flowchart of participant selection for the present study

Participant flow throughout the trial is shown in Fig. 2. Based on the screening survey, 181 respondents were screened, 180 of whom were deemed to be eligible. However, five participants withdrew before completing the baseline assessment, resulting in 175 being enrolled (intervention group: *n* = 89; control group: *n* = 86).

All 89 participants allocated to the intervention group completed the 8-week program; among these participants, 84 (94%) attended at least four of the six remote exercise classes. Two participants (intervention group: *n* = 1; control group: *n* = 1) withdrew before the follow-up assessment. Ultimately, 173 participants completed the study (intervention group: *n* = 88; control group: *n* = 85), with an overall retention rate of 98%–99% across study arms. Notably, all participants who completed the study met the accelerometer wear-time criteria, ensuring valid PA data collection as defined in the Methods. We had no reports of any participants or instructors experiencing any harm from their participation in this study.

### Demographic data

The characteristics of the participants are shown in Table 1. No significant differences in baseline variables were found between groups, except for the number of months since childbirth, which was significantly longer in the intervention group (*p* = 0.035). The mean age of participants was 34.5 years. A majority of participants held a university degree (92%, *n* = 161). Most participants were married (99%, *n* = 171), and not working (93.2%, *n* = 150) at the time of enrollment. The mean time since childbirth was 4.0 ± 1.3 months, and the mean body mass index was 21.7 ± 2.7 kg/m^2^.

**Table 1:** Characteristics of participants of the intervention and control group at baseline

Demographic variable		All	Intervention	Control	
		*n* = 175	*n* = 89	*n* = 86	*P-value*
Age (years)		35.4 ± 4.2	34.9 ± 4 7	34.0 ± 3.7	0.173
Average month since childbirth		3.6 ± 0.1	3.8 ± 0.1	3.4 ± 0.1	0.035
BMI^a^ (kg/m2)		21.7 ± 2.7	21.8 ± 2.9	21.6 ± 2.5	0.941
		n (%)	n (%)	n (%)	
Parity	Primipara	98 (56.0)	50 (56.2)	48 (55.8)	0.961
	Multiparous	77 (44.0)	39 (43.8)	38 (44.2)	
Marital statas	Married	173 (98.9)	87 (97.8)	86 (100)	0.498
	Single, Divorced	0 (0)	0 (0)	0 (0)	
	No answer	2 (1.1)	2 (2.2)	0 (0)	
Education (% College Grad)	College, University	161 (92.0)	80 (89.9)	81 (94.2)	0.216
	Junior high school, High school	8 (9.0)	5 (5.8)	13 (7.4)	
	No answer	1 (0.6)	1 (1.1)	0 (0)	
Household income^b^	More than 7 million yen	120 (68.6)	59 (66.3)	61 (70.9)	0.484
	Less than 7 million yen	47 (26.9)	24 (27.0)	23 (26.7)	
	No answer	8 (4.6)	6 (6.7)	2 (2.3)	
Occupation	Worker^c^	10 (5.7)	7 (7.9)	3 (3.5)	0.567
	Non-workerd	150 (93.2)	82 (91.0)	86 (95.3)	
	Other	2 (1.1)	1 (1.1)	1 (1.2)	
Delivery mode	Vaginal	142 (79.3)	74 (80.4)	68 (78.2)	0.707
	Casarean section	37 (20.7)	18 (19.6)	19 (21.8)	

Data are mean ± SD or n (%)

^a^BMI: Body Mass Index

^b^Household income: the average household income among individuals aged 30–39 years in Japan was 6.05 million yen (Comprehensive Survey of Living Conditions, 2024)

^c^Worker: include part-time job and school attendance; dNon-worker: include full-time homemakers and workers on a child-care leave

### Intervention effects on outcome variables

Table 2 presents the results of the baseline and 8-week follow-up assessments, including the mean changes and results of the GEE models adjusting for age and baseline values.

**Table 2:** Changes in MVPA, step counts, and psychosocial outcomes from baseline to 8-week follow-up in the intervention and control groups

Outcome Variables	Baseline		8-week follow-up		Mean change		Group difference^a^		
	Intervention	Control	Intervention	Control	Intervention	Control	Estimate (B)	95% CI	*P-value*^b^
	*n* = 88	*n* = 85	*n* = 88	*n* = 85	*n* = 88	*n* = 85			
	mean ± SD		mean ± SD		mean ± SD				
MVPA, min/day	51.6 ± 21.1	50.1 ± 27.0	60.0 ± 22.2	52.5 ± 28.8	7.6 ± 18.6	2.4 ± 13.4	5.97	1.34, 10.6	0.012
MVPA, METs/day	3.0 ± 1.4	3.0 ± 1.7	12.9 ± 12.3	8.8 ± 11.2	10.2 ± 12.4	5.8 ± 10.7	4.06	0.63, 7.49	0.02
Steps counts, steps/day	6148.5 ± 2026.5	5982.9 ± 2353.6	6812.9 ± 2196.8	6087.8 ± 2399.4	710.7 ± 1894.1	105.2 ± 1706.8	576.06	73.06, 1079.06	0.025
SF-12v2 summary score									
PCS	44.7 ± 8.2	45.1 ± 10.9	44.0 ± 8.3	44.2 ± 10.6	−0.5 ± 9.2	−0.9 ± 8.9	0.04	−2.28, 2.36	0.973
MCS	54.0 ± 7.5	52.2 ± 7.6	55.8 ± 6.9	53.6 ± 7.8	2.3 ± 7.3	1.4 ± 7.2	1.19	−0.67, 3.04	0.209
SOC	63.1 ± 12.6	61.3 ± 12.8	65.0 ± 11.6	59.5 ± 12.3	1.7 ± 9.8	−1.8 ± 8.4	4.14	1.70, 6.58	<.001
Self-efficacy for exercise									
Balance score(pros-cons)	18.3 ± 10.3	16.8 ± 10.2	19.9 ± 9.3	16.1 ± 10.9	1.9 ± 7.3	−0.7 ± 7.1	2.74	0.71, 4.78	0.008
Pros	41.5 ± 5.2	40.9 ± 5.5	41.8 ± 4.8	41.1 ± 5.4	0.4 ± 4.8	0.3 ± 4.0	0.35	−0.80, 1.50	0.554
Cons	23.1 ± 6.9	24.1 ± 6.5	21.9 ± 6.1	25.0 ± 7.2	−1.5 ± 4.8	1.0 ± 4.6	−2.46	−3.80, −1.12	<.001

Absolute values are presented in Data are mean ± SD and relative change in exponential beta coefficient (95% confidence interval)

*Abbreviations*: *MVPA* moderate-to-vigorous physical activity, *95%CI* 95% confidence interval, *SF-12v2* the Short-Form 12-Item Survey-version 2, *PCS* physical component summary scale, *MCS* mental component summary scale, *SOC* sence of coherence, *Pros* perceived positive of exercise, *Cons* negative aspects of exercise

^a^General estimating equation models (GEE) adjusted for age and baseline scores

^b^p between group comparison (group*time interaction)

### Physical activity outcomes

From baseline to follow-up, the intervention group showed significantly greater improvements in MVPA duration and volume, as well as step count, compared with the control group.

MVPA increased by 7.6 min/day in the intervention group and by 2.4 min/day in the control group. The between-group difference was 5.97 min/day ([95% confidence interval (CI): 1.34, 10.60], *p* = 0.012). In terms of MVPA volume, the intervention group increased by 10.2 METs/day, whereas the control group showed only a minimal increase of 5.8 METs/day. The between-group difference was 4.06 METs/day ([95% CI: 0.63, 7.49], *p* = 0.020). The daily step count increased significantly, by 710.7 steps/day, in the intervention group, compared with an increase of 105.2 steps/day in the control group. The between-group difference was 576.06 steps/day ([95% CI: 73.06, 1079.06], *p* = 0.025).

### Health-related quality of life

The SF-12v2 PCS score showed a nonsignificant decrease of –0.5 points in the intervention group and –0.9 points in the control group. The between-group difference was not significant (0.04 [95% CI: –2.28, 2.36], *p* = 0.973). Similarly, the MCS score increased by 2.3 points in the intervention group and 1.4 points in the control group, with no significant group difference (1.19 [95% CI: –0.67, 3.04], *p* = 0.209).

### Sense of coherence

SOC increased by 1.7 points in the intervention group, whereas it decreased slightly (–1.8 points) in the control group. The between-group difference in change was statistically significant (4.14 [95% CI: 1.70, 6.58], *p* < 0.001).

### Self-efficacy for exercise

The balance score (pros minus cons) increased by 1.9 points in the intervention group and decreased by 0.7 points in the control group, with a significant between-group difference in change (2.74 [95% CI: 0.71, 4.78], *p* = 0.008). Regarding subscales, perceived barriers to exercise (cons) decreased significantly in the intervention compared with the control group (–2.46 [95% CI: –3.80, –1.12], *p* < 0.001), whereas perceived benefits (pros) showed no significant between-group difference (0.35 [95% CI: –0.80, 1.50], *p* = 0.554).

## Discussion

The present RCT evaluated the efficacy of an 8-week remote, group-based PA intervention for postpartum women. The intervention produced significant improvements in objectively measured MVPA. In addition, favorable changes were observed in daily step count, SOC, and exercise-related self-efficacy compared with the waitlist control group. No significant between-group differences in HRQoL scores were observed.

### Feasibility and acceptability

The present intervention demonstrated excellent feasibility, with retention rates of 98%–99%, exceeding those reported in comparable technology-based interventions for mothers (86%–95%) [54, 55]. The fully remote design minimized participant burden by enabling online recruitment, data collection, and program delivery, allowing mothers to participate without leaving home and accommodating childcare demands. The high retention rate suggests that this approach is both practical and acceptable for postpartum women, supporting the scalability of web-based PA programs.

### Physical activity outcomes

Participation in the intervention significantly increased MVPA duration (by approximately 6 min/day) and daily step count (by over 570 steps/day), representing meaningful behavioral changes for this population. When interpreted in relation to current PA guidelines for pregnant and postpartum women, the observed increase of approximately 6 min per day (42 min per week) corresponds to nearly 28% of the recommended weekly MVPA volume. These findings contrast with prior systematic reviews reporting null or inconsistent effects of postpartum PA interventions on objectively measured activity levels [15, 26]. Previous RCTs of digital PA interventions in postpartum populations have used various digital modalities, including mobile apps [32, 55, 56], exercise videos [54, 56, 57], and Facebook groups [58]. For example, in a pilot study with 35 participants, Tinius et al. [32] found no differences in MVPA (device-measured and self-reported) at 12 weeks postpartum between participants in the control group and those with access to a mobile app that provided PA tracking, exercise videos, and educational content. By contrast, Mascarenhas et al. [57] reported that a group exercise intervention delivered via videoconferencing and mobile apps was effective at increasing self-reported PA among inactive mothers and was both feasible and acceptable. The present RCT adds evidence that combining synchronous group-based exercise sessions with home-based activities and digital engagement tools can effectively increase PA in postpartum women. The provision of weekly instructor-led sessions likely facilitated accountability and peer interaction, while goal setting, self-monitoring, and motivational feedback—designed to enhance autonomy, competence, and self-efficacy in accordance with self-determination theory and social cognitive theory [16, 33, 34, 59]—helped sustain engagement. These results extend the findings of Cramp and Brawley [37], who reported benefits from an in-person group-mediated intervention, by showing that comparable improvements can be achieved in a fully remote format, thereby broadening access for women facing logistical and childcare barriers. A methodological consideration concerns the use of MET-based cut points derived from the Active Style Pro algorithm, which was validated in adult populations. Pregnancy and the early postpartum period involve physiological adaptations—including changes in body composition [60, 61] and lactation-related energy expenditure [62]—that may influence resting metabolic rate and affect the interpretation of MET-based intensity estimates [62]. Therefore, applying adult-derived thresholds may result in some misclassification of absolute intensity in postpartum women. Such misclassification would likely be non-differential with respect to group allocation and thus bias estimates toward the null rather than inflate intervention effects. In the absence of validated postpartum-specific accelerometer cut points, we applied the device’s standard adult thresholds to maintain comparability with current guidelines and prior research.

### Psychosocial outcomes: HRQoL, self-efficacy, and SOC

No significant improvements in PCS or MCS scores on the SF-12v2 were observed. The absence of any significant change may reflect the relatively short intervention duration, modest frequency of supervised sessions, and limited statistical power to detect changes in global HRQoL. Prior studies have similarly reported mixed findings. Haruna et al. [63] found improvements in selected SF-36v2 subscales, but not in summary scores, and Sanchez-Garcia et al. [64] observed MCS improvements following a longer program (12 weeks) with higher exercise frequency. Together, these results suggest that more intensive or prolonged exercise interventions are needed to elicit clinically meaningful improvements in HRQoL. The mean PCS values in our sample were below the 25th percentile for age-matched Japanese women (interquartile range: 47.1–54.3), indicating lower baseline physical health status. Our study contributes to this evidence base by highlighting the need for dose–response trials to determine not only the optimal intensity, frequency, and duration of postpartum exercise programs, but also how these parameters can be tailored to individual baseline physical health status to maximize HRQoL benefits.

A key strength of the intervention was its effect on exercise-related self-efficacy, primarily driven by reductions in perceived barriers. The observed shift in decisional balance suggests that the intervention successfully reduced obstacles to PA while simultaneously reinforcing positive perceptions of exercise. In a US cohort, the most frequently reported barriers to PA at both 3 and 12 months postpartum were a lack of time and challenges related to childcare [4]. Remote participation reduced time and childcare constraints, and exercise routines were designed to allow infant interaction, which may have enhanced motivation among women with limited prior exercise habits. For some participants, the opportunity for physical contact and playful interaction with their child may have been as important a motivator as the exercise itself. These findings align with studies identifying intrapersonal barriers as the most salient impediments to postpartum PA [4, 65] and demonstrate that remote group formats can mitigate these barriers while simultaneously strengthening positive attitudes toward exercise. Improved self-efficacy is a well-established predictor of long-term exercise maintenance, suggesting that this approach may foster sustained PA engagement [66].

Significant improvements in SOC were also observed, suggesting that structured PA programs may influence psychological resources relevant to coping and resilience. Although SOC has traditionally been considered a relatively stable construct in adulthood, emerging evidence indicates that it can be strengthened through structured PA programs. Intervention studies among older adults have shown that baseline SOC levels [67], as well as the duration and frequency of the intervention [68], influence the effectiveness of PA on SOC. Thompson et al. [68] found that in an 18-month community-based PA intervention, SOC was strengthened only among participants with low baseline SOC. Kekäläinen et al. [67] reported that in a 9-month resistance training program, SOC showed a nonsignificant increase at 3 months but significantly improved at 9 months among participants training twice weekly compared with those training once weekly or controls. In the present study, participants’ baseline SOC levels were already higher than the mean SOC reported for the general female population in Japan aged 35–44 years (mean 56.7, SD 12.2) [50], and the intervention period was relatively short. Nevertheless, SOC increased significantly. This finding suggests that even in populations without markedly low SOC, structured PA may contribute to strengthening this salutogenic resource, which represents a novel finding. Participants frequently cited the value of peer support and shared experiences in sustaining their participation. Group-based remote exercise may strengthen the core components of SOC—comprehensibility, manageability, and meaningfulness—by providing structured activity, social connectedness, and a sense of mastery.

### Strengths and limitations

This study has several limitations. First, the participants were self-selected volunteers; therefore, the results may not be representative of postpartum women who are less motivated to engage in exercise. Second, the participants tended to have higher income and educational levels than the general population and to reside in urban areas, which may have introduced selection bias. Third, the participants in this study may have had relatively low barriers to online program participation and a higher level of digital competency. Fourth, we evaluated changes only between preintervention and postintervention assessments and were unable to examine the program’s long-term effects on PA. Fifth, self-reported PA data were not standardized, limiting quantitative comparison with accelerometer-based measures. Future research should investigate dose–response relationships and long-term effects to optimize the program design for diverse postpartum populations. Despite these limitations, this study also has notable strengths. To our knowledge, this is the first remote PA intervention study conducted among postpartum women in Japan. Another strength is the high class attendance rate (94%), likely facilitated by the online format, which enabled the participants to attend supplementary lectures even if they had scheduling conflicts and to continue participation more easily while caring for their infants.

## Conclusions

This 8-week remote, group-based PA intervention effectively increased objectively measured MVPA, daily step counts, exercise-related self-efficacy, and SOC among postpartum women. Designed to accommodate the lifestyle demands and constraints of the postpartum period, the intervention integrated group-based exercise with behavioral strategies was grounded in self-determination theory and social cognitive theory. Through instructor-led online sessions and home-based participation using exercise videos, the program supported autonomy, competence, and peer connectedness while facilitating engagement in PA. By promoting accessible and sustainable PA participation in a population at risk of inactivity, the present intervention may be expected to contribute to improvements in physical and mental health through strengthened self-efficacy and psychological resilience.

## Supplementary Information

Supplementary Material 1.

Supplementary Material 2.

Supplementary Material 3.

Supplementary Material 4.

## Data Availability

Due to confidentiality and restrictions being imposed by the research ethics committee of Chiba Institute of Technology, the data used in this study have not been deposited in a public repository. The individual responsible for data management is Dr. Yumi Nomura. Data inquiries should be sent by email (yumi.nomura@chibatech.ac.jp).

## Abbreviations

PA: Physical activity
MVPA: Moderate-to-vigorous physical activity
RCT: Randomized controlled trial
HRQoL: Health-related quality of life
SOC: Sense of coherence
MET: Metabolic equivalent
SF-12v2: Short Form 12-Item Survey version 2
PCS: Physical component summary
MCS: Mental component summary
GEE: Generalized estimating equation
CI: Confidence interval
